# The role of social relatedness and self-beliefs in social functioning in first-episode psychosis: Are we overestimating the contribution of illness-related factors?

**DOI:** 10.1192/j.eurpsy.2020.90

**Published:** 2020-10-09

**Authors:** César González-Blanch, Leonardo A. Medrano, Sarah Bendall, Simon D’Alfonso, Daniela Cagliarini, Carla McEnery, Shaunagh O’Sullivan, Lee Valentine, John F. Gleeson, Mario Alvarez-Jimenez

**Affiliations:** 1 Mental Health Centre, University Hospital Marqués de Valdecilla – IDIVAL, Santander, Spain; 2 Faculty of Psychology, Pontificia Universidad Católica Madre y Maestra, Santiago de los Caballeros, Dominican Republic; 3 Orygen, Melbourne, Victoria, Australia; 4 Centre for Youth Mental Health, The University of Melbourne, Melbourne, Victoria, Australia; 5 School of Computing and Information Systems, University of Melbourne, Parkville, Victoria, Australia; 6 Healthy Brain and Mind Research Centre and School of Behavioural and Health Sciences, Australian Catholic University, Melbourne, Victoria, Australia

**Keywords:** Cognition, negative symptoms, self-efficacy, social support, structural equation modeling

## Abstract

**Objective:**

Numerous research studies have demonstrated an association between higher symptom severity and cognitive impairment with poorer social functioning in first-episode psychosis (FEP). By contrast, the influence of subjective experiences, such as social relatedness and self-beliefs, has received less attention. Consequently, a cohesive understanding of how these variables interact to influence social functioning is lacking.

**Method:**

We used structural equation modeling to examine the direct and indirect relationships among neurocognition (processing speed) and social cognition, symptoms, and social relatedness (perceived social support and loneliness) and self-beliefs (self-efficacy and self-esteem) in 170 individuals with FEP.

**Results:**

The final model yielded an acceptable model fit (*χ* ^2^ = 45.48, comparative fit index = 0.96; goodness of fit index = 0.94; Tucker–Lewis index = 0.94; root mean square error of approximation = 0.06) and explained 45% of social functioning. Negative symptoms, social relatedness, and self-beliefs exerted a direct effect on social functioning. Social relatedness partially mediated the impact of social cognition and negative symptoms on social functioning. Self-beliefs also mediated the relationship between social relatedness and social functioning.

**Conclusions:**

The observed associations highlight the potential value of targeting social relatedness and self-beliefs to improve functional outcomes in FEP. Explanatory models of social functioning in FEP not accounting for social relatedness and self-beliefs might be overestimating the effect of the illness-related factors.

## Introduction

Subsequent to experiencing a first-episode psychosis (FEP), many individuals show—regardless of whether or not the symptoms remit—remarkably stable long-term impairments in major areas of everyday life [1,2]. Functional impairments in various areas—including work or education, interpersonal relations, and self-care—are associated with high costs of care and decreased quality of life [3]. Consequently, these areas represent a crucial therapeutic goal for people with FEP. However, the effects of treatments on functional outcomes have been somewhat neglected compared with symptom-based outcomes [4].

Based on a systematic review and meta-analysis, we have previously suggested that novel treatments targeting cognitive deficits may improve functional outcomes in FEP [5]. However, a recent meta-analysis [6] showed only small-to-medium effect sizes for the relationship of neurocognition and social cognition with functional outcomes, explaining 9% of the total average variance; thus, a significant proportion of the variance remains unexplained.

To date, research carried out to identify the determinants of functioning in individuals with psychotic disorders has focused mainly on illness-related variables that are considered to have a biological origin, such as cognitive impairment or symptom severity [7,8]. However, according to the biopsychosocial perspective, two other major areas have also been targeted: (a) psychological and personal resources, such as self-efficacy or coping skills and (b) social factors, such as social support or the impact of episodic stressors [9], although most studies conducted to date have considered these factors separately. Consequently, a cohesive understanding of how these three major domains might interact to influence functional outcomes in these in affected individuals is lacking.

In contrast to the clinical focus on deficits, consumer perspectives have focused on the positive process of recovery from psychosis, including an emphasis upon the individual’s appraisal of personal and social resources [10]. This recovery model assumes that all individuals have the capacity to improve and develop a life that is distinct from their illness. The renewed focus on social recovery is also consistent with recent psychological theories and treatments, which have proposed self-constructs and positive emotions as important targets to promote social functioning in psychosis [11–14].

People with psychotic disorders diagnosis may be particularly vulnerable to social isolation (i.e., loneliness), which refers to the discrepancy between the desired and achieved number and quality of the relationships, and more likely to perceive the support received from others and the quality of this support (i.e., perceived social support) as lower than the general population [15]. In the prevailing vulnerability-stress models describing the course of schizophrenia, social support has been postulated as a key environmental protective factor [16,17]. Nevertheless, the relationship between social support, loneliness, and functioning in psychotic disorders remains largely unexplored [18,19].

According to the published literature, judgments about one’s own worth and capabilities (such as self-esteem and self-efficacy) are potential factors that may directly or indirectly influence social functioning. Indeed, several studies have found links between self-esteem (i.e., one’s sense of worth or value) and social functioning in chronic schizophrenia [20–22]. The relationship of self-esteem with psychotic phenomena is more controversial, with some studies suggesting that negative self-esteem is associated with more paranoia in response to social stressors [23], whereas others have found instead an association between change in self-esteem and change in negative symptoms but only during the early stages of treatment [24]. Interestingly, the relationship between self-esteem and psychosocial functioning may be moderated by neurocognitive functioning [25]. Some studies have found that lower self-efficacy (i.e., one’s confidence in the ability to perform behaviors necessary to produce specific performance attainments) is associated with poorer outcomes in terms of psychosocial functioning, negative symptoms, and neurocognition [26–29] but there is still no clear support for the mediational role of self-efficacy [27,30,31]. To the contrary, several studies have suggested that negative symptoms could mediate the impact of self-efficacy on functioning [26,28,30].

The interactions between the variables described above are likely to be complex and reciprocal. To examine the relationships between social relatedness (i.e., the need to feel connected and to perceive support from others) and self-beliefs (i.e., the subjective evaluation of our own worth and capabilities), cognition, symptoms, and functioning, we conducted a secondary analysis of cross-sectional data collected in a randomized controlled trial (RCT) carried out to improve long-term social functioning in early psychosis [11]. Based on findings reported in previous studies [6,11,18,19,26,28] and on theoretical speculation, we hypothesized that (a) neurocognition will have an indirect effect on functioning through social cognition, (b) positive and negative symptoms will exert a direct effect on social functioning and will partially mediate the effect of social cognition on social functioning, and (c) social relatedness (perceived social support and loneliness) and self-beliefs (self-efficacy and self-esteem) will have a direct impact on social functioning and will mediate the effect of social cognition on social functioning in patients with FEP.

## Methods

### Participants

In the present study, we analyzed baseline data obtained from all participants (*N* = 170) in the HORYZONS trial [11], which we considered as a single cohort. The HORYZONS RCT was conducted to evaluate the effectiveness of an online intervention designed to extend the benefits of specialized FEP services. The study inclusion criteria were (a) a first episode of a psychotic disorder or a mood disorder with psychotic features according to the Diagnostic and Statistical Manual of Mental Disorders (DSM-IV) criteria, (b) age 16–27 years, inclusive, (c) ≤ 6 months of treatment with an antipsychotic medication prior to registration with Early Psychosis Prevention and Intervention Centre (EPPIC), and (d) remission of positive symptoms of psychosis, defined as ≥4 weeks of scores ≤3 (mild) on items P2 (conceptual disorganization) and G9 (unusual thought content) on the Positive and Negative Syndrome Scale (PANSS) [32] and scores ≤4 (moderate) with no functional impairment on items P3 (hallucinatory behavior) and P1 (delusions) on the PANSS. Participants were included in the HORYZONS trial at the point of discharge from EPPIC, a specialist FEP service, Melbourne (Australia). Hence, the FEP sample included in the current study represented young people in clinical remission with a broad range of DSM-IV affective and nonaffective psychotic disorders who have received treatment in line with the mainstream model of early intervention for psychosis (including pharmacological treatment and a range of psychosocial interventions). Exclusion criteria were (a) severe intellectual disability and (b) inability to speak or read English. Additional exclusion criteria to ensure safety within the online system included a DSM-IV diagnosis of antisocial or borderline personality disorder.

All participants provided written informed consent. Ethics approval for the trial was provided by the Melbourne Health Research and Ethics Committee (No. 2013.146).

### Assessments

#### Functioning

The Personal and Social Performance Scale (PSP) [33], a clinician-rated instrument, was used to evaluate overall social functioning. The PSP measures four areas of social and individual performance (self-care, socially useful activities—including work and study—personal and social relationships, and disturbing and aggressive behaviors) and provides a global score ranging from 1 to 100, with higher scores representing better functioning.

#### Social relatedness

The 2-Way Social Support Scale (2-Way SSS) [34], a 21-item self-report measure, was used to measure perceptions of giving and receiving emotional and instrumental social support; higher scores are reflective of greater perceived social support. Subjective feelings of loneliness were assessed by the UCLA Loneliness Scale, version 3 (UCLA-3) [35], a 20-item self-report measure in which scores range from 20 (little loneliness) to 80 (great loneliness).

#### Self-beliefs

Self-efficacy was measured by the Mental Health Confidence Scale (MHCS) [36], a 16-item self-report questionnaire; the total score ranges from 16 to 96, with higher scores indicating greater self-efficacy. Self-esteem was measured by the Self-Esteem Rating Scale-Short Form (SERS-FS) [37], a 20-item self-report scale comprised of two subscales to separately assess positive and negative self-esteem. For the purposes of this study, we used the total score, which was highly correlated in our study with both subscales (*r* > 0.85). Higher scores of the total score correspond to more positive self-esteem.

#### Positive and negative symptoms

Two subscales of the PANSS were used to assess negative (e.g., apathetic social withdrawal and blunted affect) and positive (e.g., hallucinatory behavior and delusions) symptoms [32]. Each subscale contains seven items ranging from 1 to 7, with higher scores indicating greater symptom severity.

#### Cognition

The Digit Symbol Substitution Test was used to assess neurocognitive deficits, which taps an information processing inefficiency that is a central feature of the cognitive deficit in schizophrenia and FEP [38,39]. This test consists of a pen-and-paper task containing rows of small blank squares, each paired with a randomly assigned number from 1 to 9. Above these rows is a printed reference key that pairs each number with a unique symbol. Using the key provided, the examinee has 120 s to fill in the corresponding symbol for each number. A higher score indicates better cognitive performance.

Two social cognition domains were assessed: (a) emotion processing and (b) theory of mind (ToM). The Bell Lysaker Emotion Recognition Task (BLERT) [40] was used to assess emotion processing. This task consists of 21 ten-second video clips of a male actor portraying different emotions, and the participants are required to identify the emotion expressed by the actor. The total score ranges from 0 to 21, based on the total number of correctly identified emotions. The Hinting Task [41] was used to evaluate ToM. This task consists of 10 short written passages presenting an interaction between two characters. The task is designed to measure the ability to infer true intent behind indirect speech utterances. Total score ranges from 0 to 20, with higher scores indicating more appropriate responses.

### Statistical analyses

As a preliminary analysis, we examined the pairwise correlation and covariance matrix for the study variables. Given that this study relies on self-reported data, we tested for possible bias due to common method variance using Harman’s single factor test [42].

Structural equation modeling (SEM) was used to examine the relationships among variables. The main advantage of the SEM technique is that it allows for a more precise analysis of empirical data by taking into account latent variables and complex patterns of relationships between variables [43]. SEM also allows for the simultaneous use of several variables for a theoretical, unobservable construct, which ultimately leads to more valid conclusions on the construct level, helping to reduce measurement error. In the present study, social relatedness and self-beliefs were defined as latent variables. Scores on the 2-Way SSS and UCLA-3 tests were used to assess social relatedness, while scores on the MHCS and SERS-FS were used for self-beliefs. Social cognition was also defined as a latent variable, based on the Hinting Task and BLERT scores.

Social relatedness, self-beliefs, cognition (processing speed and social cognition), and (positive and negative) psychotic symptoms were included in our initial SEM model (see the initial model tested inFigure 1). The final model was obtained by removing nonsignificant effects. Since two variables may be connected in a SEM through several different pathways, direct, indirect, and total effects were estimated. Squared multiple correlations (*R* ^2^) were obtained for each endogenous variable to estimate the amount of variance explained by its predictor. Several goodness-of-fit-indices were calculated to examine the overall model fit, as follows: the absolute fit index (*χ* ^2^); the goodness of fit index (GFI); the Tucker–Lewis index (TLI); the comparative fit index (CFI); and the root mean square error of approximation (RMSEA). GFI, TLI, and CFI values >0.90 and RMSEA values <0.08 indicated acceptable model fit, while values >0.95 (GFI, TLI, and CFI) and <0.05 (RMSEA) are indicative of excellent fit [44]. All models were estimated using Mplus 7.11 [45] with a robust weighted least square (WLSMV) estimator. Previous research has shown that WLSMV results in a more accurate estimation of key model parameters than other methods [46,47]. In addition, simulation studies have shown that WLSMV performs well even with sample sizes less than 200 cases [48].

**Figure 1.**
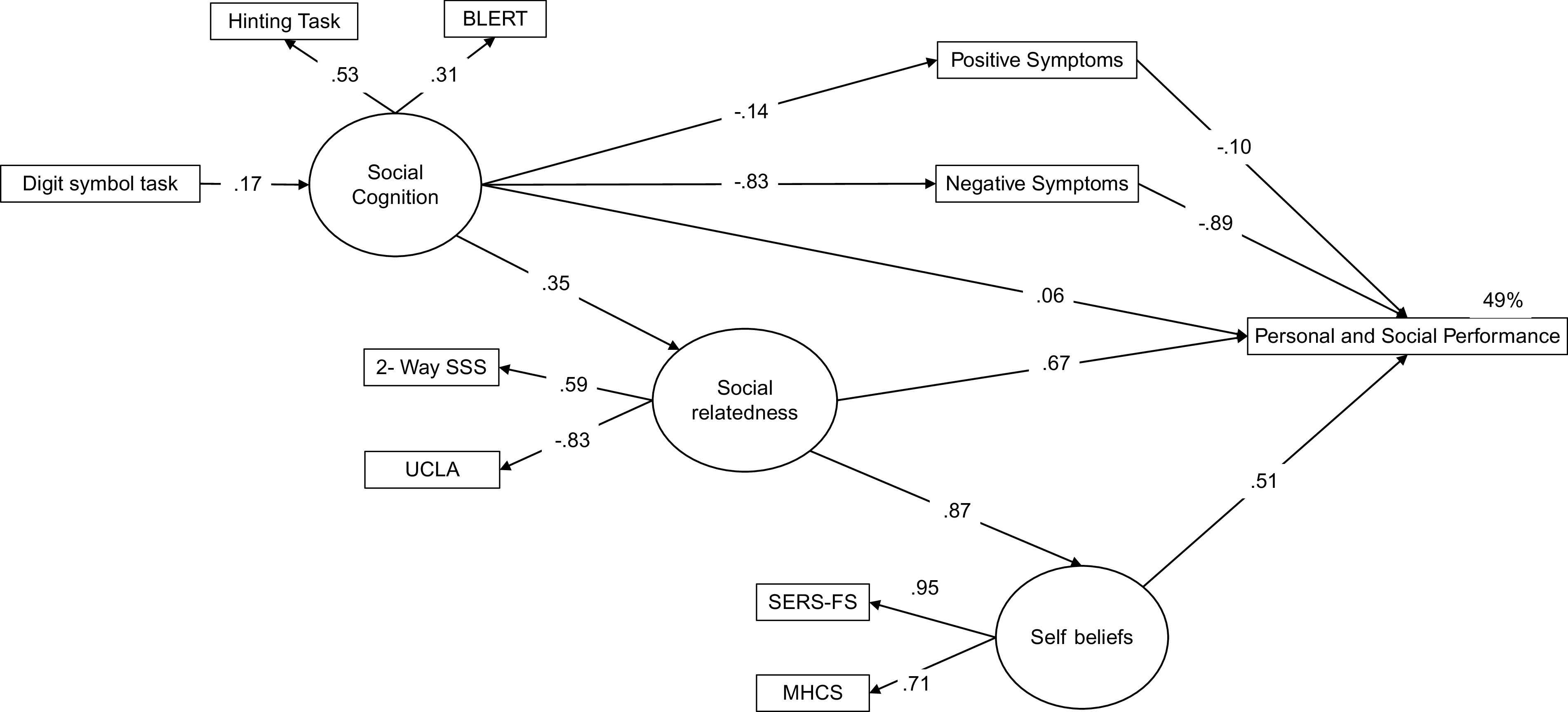
Initial structural equation model. Rectangles represent observed measured variables. Circles represent unobserved latent variables. Values are standardized path coefficients. The squared multiple correlation value (*R* ^2^) of the Personal and Social Performance Scale indicates the amount of variance explained by its predictors. PANSS, Positive and Negative Syndrome Scale; BLERT, Bell Lysaker Emotion Recognition Task; UCLA-3, UCLA Loneliness Scale Version 3; MHCS, Mental Health Confidence Scale; 2-Way SSS, 2-Way Social Support Scale; SERS-FS, Self-Esteem Rating Scale-Short Form.

## Results

### Characteristics of the sample

Of the 170 participants in the study, 90 (53%) were male. The mean age of the sample at intake was 20.9 years (SD = 2.9). The average level of education was 11.1 years (SD = 1.1). The median duration of untreated psychosis (DUP) was 30 days. The baseline characteristics of the sample are summarized inTable 1. Pearson correlations between the study variables are presented in Table S1.

**Table 1.** Characteristics of the study sample (*N* = 170).

Variables of interest	Mean (SD)	Min/Max
Age	20.9 (2.9)	16/27
Gender, male, *n* (%)	90 (52.9)	
Education years	11.1(1.1)	8/12
DUP^a^	181.2 (375.4)	0/2555
Diagnosis, *n* (%)		
Schizophrenia	29 (17.1)	
Schizoaffective disorder	19 (11.2)	
Schizophreniform and brief psychotic disorder	21 (12.4)	
Bipolar disorder	35 (20.6)	
Depression with psychotic features	23 (13.5)	
Delusional disorder	5 (2.9)	
Substance-induced psychotic disorder	8 (4.7)	
Psychotic disorder NOS	30 (17.6)	
PSP	66.5 (13.2)	32/95
PANSS positive	10.1 (3.2)	7/25
PANSS negative	11.2 (3.6)	7/24
Hinting Task	16.2 (2.6)	8/20
BLERT	16.4 (2.7)	7/21
Digit Symbol	7.9 (2.4)	3/14
UCLA-3	46.8 (11.2)	23/76
MHCS	66.7 (14.9)	24/96
2-Way SSS	75.1 (17.4)	17/100
SERS-FS	10.7 (22.8)	−47/57

Abbreviations: BLERT, Bell Lysaker Emotion Recognition Task; MHCS, Mental Health Confidence Scale; NOS, not otherwise specified; PANSS, Positive and Negative Syndrome Scale; PSP, Personal and Social Performance scale; SERS-FS, Self-Esteem Rating Scale-Short Form; UCLA-3, UCLA Loneliness Scale Version 3; 2-Way SSS, 2-Way Social Support Scale.

a Median duration of untreated psychosis (DUP) was 30 days.

### Initial data exploration

To detect atypical cases on the analyses, *z*-scores were calculated for each item and checked for outliers (range, *z* ± 3), and the Mahalanobis distance statistical procedure (*D* ^2^) was applied. A total of 46 univariate and 7 multivariate atypical cases were identified. To determine if these atypical cases had an impact on the correlation coefficients, bivariate correlation matrices were calculated with and without the atypical cases. Finally, Cohen’s *q* [49] was calculated to determine if there were any relevant differences between the *r* values. All *q* values were less than 0.10, indicating a very small effect size. Based on this, we decided to retain the atypical cases for the analysis.

Missing data accounted for less than 5% of the whole dataset, and these data were missing completely at random (MCAR) according to Little’s MCAR test (*χ* ^2^ = 93.587, df = 105, *p* = 0.780); as a result, we used listwise deletion. Asymmetry and kurtosis descriptive statistics showed that the distribution for all variables was close to normal, based on *a* ± 2 range of values [50] (seeTable 1). Multivariate normality was verified using Mardia’s coefficient, which yielded a value (19.3) well below the critical value of 70 [51].

### SEM analyses

For the SEM analyses, we first performed Harman’s single factor test, the results of which revealed a poor fit to the data: *χ* ^2^ (44) = 143.59; GFI = .81; CFI = .77; TLI = .71; RMSEA = .13. This test indicated that common method variance was not a serious concern in our analyses. Next, we tested the hypothesized model (Figure 1), with the results indicating that the model presented acceptable fit: (*χ* ^2^ (38) = 73.25; CFI = 0.92; GFI = 0.91; TLI = 0.90; RMSEA = 0.08, 90% confidence interval [CI] 0.06 to 0.10).

To specify the final model, we removed the paths that did not show a significant effect and analyzed the modification indices. Specifically, positive symptoms were removed from the model and a new path was specified between the negative symptoms and social relatedness. This model accounted for 45% of variance of PSP; it was more parsimonious and proved to have a better fit than the initial model (*χ* ^2^(38) = 45.48; CFI = .96; GFI = .94; TLI = .94; RMSEA = .06; 90% CI 0.02 to 0.10). Standardized path coefficients are reported inFigure 2.

**Figure 2.**
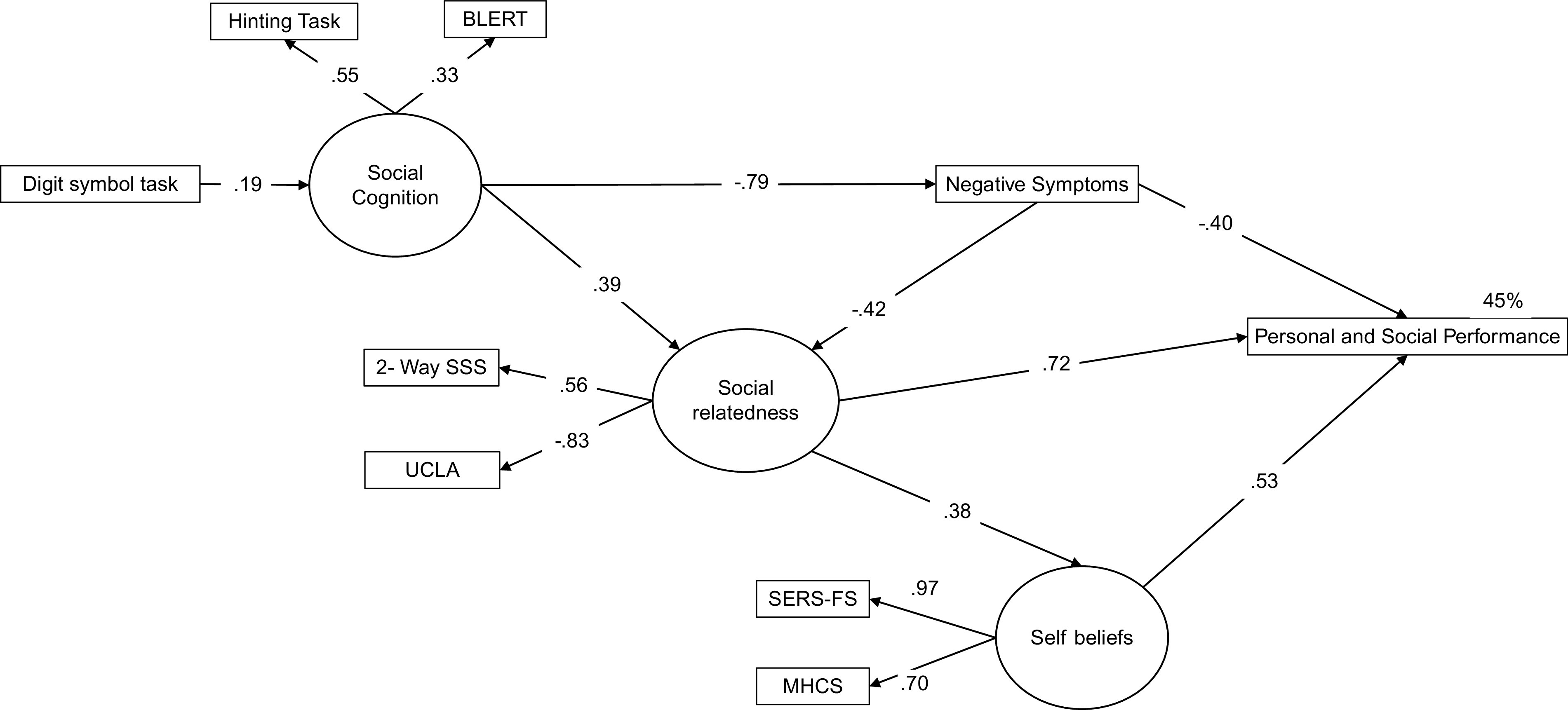
Final structural equation model. Rectangles represent observed measured variables. Circles represent unobserved latent variables. Values are standardized path coefficients. The squared multiple correlation value (*R* ^2^) of the Personal and Social Performance Scale indicates the amount of variance explained by its predictors. PANSS, Positive and Negative Syndrome Scale; BLERT, Bell Lysaker Emotion Recognition Task; UCLA-3, UCLA Loneliness Scale Version 3; 2-Way SSS, 2-Way Social Support Scale; MHCS, Mental Health Confidence Scale; SERS-FS, Self-Esteem Rating Scale-Short Form.

As shown inFigure 2, processing speed had a direct and significant contribution to social cognition, which in turn had a direct effect on negative symptoms and on social relatedness. Negative symptoms had a direct and significant contribution to PSP, indicating that higher levels of negative symptoms are associated with poorer functioning. Social relatedness had a significant direct on PSP and an indirect effect on PSP through self-beliefs. Finally, self-beliefs showed a direct effect on PSP. These results indicate that higher levels of social relatedness and self-beliefs were associated with better social functioning (see estimates of direct and indirect effects for each variable inTable 2).

**Table 2.** Estimates of direct, indirect, and total effects of illness-related and social relatedness and self-beliefs on PSP in the final model.

	Direct	Total indirect	Total
Processing speed^a^	–	0.11	0.11
Social cognition	–	0.59*	0.59*
PANSS-negative symptoms	−0.42*	−0.30*	−0.72*
Social relatedness	0.20*	0.72*	0.92*
Self-beliefs	0.53*	–	0.53*

Abbreviations: PANSS, Positive and Negative Syndrome Scale; PSP, Personal and Social Performance Scale.

a Measured by a Digit Symbol task.

* *p* < 0.001.

## Discussion

The aim of the current study was to examine the role of social relatedness and self-beliefs in explaining social functioning in a sample of individuals with FEP. The SEM approach was used to conduct the analysis, as it takes into account latent variables and complex patterns of relationships between variables. Our findings indicated that social relatedness and self-beliefs, together with social cognition and negative symptoms, explained 45% of the variance in social functioning.

Most previous research has linked, in separate studies, specific variables to diverse aspects of social functioning. For example, self-efficacy was found to be an independent predictor of functioning in FEP [28] and, in other models, the effects of self-efficacy on functioning were mediated through negative symptoms [30,31]. In our study, latent variables reflecting social relatedness and self-beliefs were linked to social functioning. Overall, this finding is consistent with research showing that functioning does not depend only on illness-related factors [52]. There is a large body of evidence indicating that perceived personal resources are linked to performance attainments [53], and some studies have confirmed this association in individuals with FEP [12,28].

Our final model showed a direct effect of social relatedness on functioning and an indirect effect through self-beliefs. Furthermore, a path was specified between negative symptoms and social relatedness, indicating that at least part of the well-recognized effect of negative symptoms on social functioning is due to its influence on loneliness and perceived lack of social support. This is consistent with data from meta-analyses showing that there is a significant relationship between loneliness and psychotic symptoms in people with psychosis [54]. Specifically, higher levels of negative symptoms have been associated with a lack of friendships in individuals with psychotic disorders [55], and low satisfaction with social support and loneliness have been associated with more negative symptoms [56]. The association between social relatedness and negative symptoms could be partly due to social anhedonia, a type of social withdrawal driven by the lack of perceived reward from social situations. However, the so-called “anhedonia paradox” posits that individuals with schizophrenia spend a smaller proportion of their time in social situations and other pleasurable activities despite their apparently normal hedonic responses [57]. A possible explanation for this is the dissociation of anticipatory (the experience of pleasure related to future activities) versus consummatory (in-the-moment pleasure experience) hedonic systems. It has been postulated that the negative symptoms could be largely related to deficits in anticipatory anhedonia. Interestingly, social functioning has been related to individuals’ reports of anticipatory but not consummatory pleasure [58].

In line with previous research [4,59–63], negative symptoms in our model had a strong and direct relationship with functioning and also influenced individuals’ social relatedness. Contrary to our hypothesis, however, positive symptoms dropped-out of the final model. A possible explanation for this is that most participants in this study were in remission of positive symptoms. Besides this, negative symptoms have been shown to be more strongly related to functioning than any of the other symptoms [7,62,64].

The relationship between neurocognition (as measured by a processing speed task) and functioning was mediated by social cognition, which is in line with previous studies [65]. However, at odds with most previous research [6], our model did not show a direct path between social cognition and functioning. Instead, the influence of social cognition on functioning was mediated by social relatedness and negative symptoms. Interestingly, in Lin and colleagues’ study, [64] the direct path from cognitive function (a latent variable which included neurocognition and social cognition variables) to functional outcome was no longer significant when symptoms were entered into the model. A possible explanation is that the unique contribution of social cognition to social functioning depends on the domains of social cognition assessed [6] as well as the constructs examined in the model. As has been suggested by Beck et al. [12], the fact that in most previous studies, psychological and social factors have typically been viewed as confounds rather than as meaningful sources of variance might have led to an overestimation of the magnitude of the effect of neurocognition and social cognition on functioning. This is consistent with earlier research that overestimated the effect of neurocognition on functioning because the role of social cognition was not taken into account (e.g., [65]).

It is worth noting that the proportion of the variance of social functioning explained by our model is greater than that found in other cross-sectional studies using path analysis or SEM with a more restricted set of constructs related to social support and self-beliefs (variance accounted for ranges from 5 to 25%) [66–69] and more similar to other studies that have included self-efficacy (31%) [28] or a comprehensive set of illness-related variables, personal resources, and context-related factors (54%) [52].

The direct explanatory power of social relatedness and self-beliefs highlights the importance that these experiences might play in the theoretical explanation of the relationship of negative symptoms and cognition with social functioning in FEP, which is crucial for enhancing the effects of early interventions in this population. In practical terms, there is a need to address these subjective domains and not just illness-related factors such as symptoms or cognitive functioning. Furthermore, given the central role of social relatedness, it is possible that the functional benefits of social cognition interventions can be explained best by the impact of these domains on the individual’s appraisals of social relatedness. On the other hand, developing psychosocial interventions targeting personal competence and connectedness with others might be the best way to augment social functioning in young people at the early stages of a psychotic disorder [11,13,70,71].

### Limitations

This study has several limitations. First, we used a cross-sectional design, thus causality can only be inferred, and we cannot rule out the possibility of reverse causation as an explanation of our findings. Longitudinal studies are needed to determine the directionality of the investigated variables. Second, the sample size was only moderate for conducting a SEM analysis, which might have resulted in inaccurate parameter estimates and model fit statistics. However, to ameliorate this limitation, we tested the validity of the model with fit indices which are less affected by sample size (such as CFI), and we used WLSMV, which has proven to perform well with sample sizes with less than 200 cases [48]. Third, this study involves a secondary analysis of an RCT, and data were not collected to test our hypothesis; other variables potentially related to social functioning (for example, personality, motivation, childhood trauma, or socioeconomic conditions) were not included in this study. Fourth, this study uses an FEP sample with a broad range of diagnoses; given the sample size, the model could not be tested separately for affective and nonaffective psychoses. Moreover, the relevance of illness-related factors can arguably change with the progression of the illness- or treatment-related processes. However, contrary to the earliest conceptions of schizophrenia, recent meta-analytic data suggest that negative symptoms are likely to improve over time [72] and cognitive deficits in first-episode samples are comparable to those in later phases of the illness [73]. Affective psychotic disorders, however, might exhibit a different longitudinal course [74]. Hence, our findings could not be generalized to other phases of illness. Fifth, DUP, a predictor of long-term functional recovery [5], was comparatively briefer in our cohort than in the general FEP population. Finally, we used a global measure of social functioning (PSP total score), which might not be sufficiently precise to detect more specific relationships between variables. Nonetheless, the PSP has been shown to have high reliability and validity in both acutely ill [75] and stable [33] mental health patients. In addition, we used a single measure of processing speed (a Digit Symbol task) instead of a more comprehensive set of measures to assess more varied and specific neurocognitive domains. Although Digit Symbol tasks have shown to be particularly good in capturing the generalized dysfunction that may underlie widespread cognitive failures in psychotic disorders [39] and have shown the largest association with social functioning [76], it is plausible that other measures of specific cognitive domains could yield different results.

### Conclusions

This study underscores that self-beliefs and the perceptions of social connections with others in young people with FEP are related to their social functioning. Psychological therapies focused on developing positive ways of self-relating and enhancing social connections may offer a fruitful approach to improve functional outcomes in young people with psychosis. In addition, the findings of this study also suggest that both individuals’ subjective experiences and illness-related factors play complementary roles in influencing functional outcomes in FEP, and, therefore, importantly, the lack of acknowledgment of social relatedness and self-beliefs in the explanatory models of social functioning in FEP can lead to an overestimation of the effect of the illness-related factors.

## Data Availability

The data that support the findings of this study are available upon request, Prof. Mario Alvarez-Jimenez (mario.alvarez@orygen.org.au).

## Supplementary material

For supplementary material accompanying this paper visit https://doi.org/10.1192/j.eurpsy.2020.90. click here to view supplementary material
