# Supplementary material for: The role of social relatedness and self-beliefs in social functioning in first-episode psychosis: Are we overestimating the contribution of illness-related factors?
Source: Eur Psychiatry. 2020 Oct 9;63(1):e92. doi: 10.1192/j.eurpsy.2020.90 (PMC7681152; doi:10.1192/j.eurpsy.2020.90)
Supplement: Supplementary file 1 [file S0924933820000905sup001.docx]

Table S1. Intercorrelations between the variables included in the structural equation model^a^

| Variables | PSP | PANSS positive | PANSS negative | Hint task | BLERT | Digit Symbol | UCLA-3 | MHCS | 2-Way SSS |
| --- | --- | --- | --- | --- | --- | --- | --- | --- | --- |
| PSP | - |  |  |  |  |  |  |  |  |
| PANSS positive | -.358*** | - |  |  |  |  |  |  |  |
| PANSS negative | -.478*** | .252** | - |  |  |  |  |  |  |
| Hinting task | .107 | -.048 | -.434*** | - |  |  |  |  |  |
| BLERT | .063 | .023 | -.217** | .200* | - |  |  |  |  |
| Digit Symbol | .143 | .024 | -.216** | .147 | .188* | - |  |  |  |
| UCLA-3 | -.313*** | .349*** | .247** | -.076 | -.195* | -.063 | - |  |  |
| MHCS | .319*** | -.255** | -.338*** | .026 | .137 | .098 | -.656*** | - |  |
| 2-Way SSS | .380** | -.114 | -.364*** | .122 | .251** | .079 | -.495*** | .589*** | - |
| SERS-FS | .257 | -.272** | -.289*** | .042 | .076 | .088 | -.721*** | .708*** | .443*** |

PSP, Personal and Social Performance scale; PANSS, Positive and Negative Syndrome Scale; BLERT, Bell Lysaker Emotion Recognition Task; UCLA-3, UCLA Loneliness Scale Version 3; MHCS, Mental Health Confidence Scale; 2-Way SSS, 2-Way Social Support Scale; SERS-FS, Self-Esteem Rating Scale-Short Form.

^a^Pearson’s correlation coefficients.

*p < 0.05, **p < 0.01, ***p < 0.001.
